# Do Family Physicians Retrieve Synopses of Clinical Research Previously Read as Email Alerts?

**DOI:** 10.2196/jmir.1683

**Published:** 2011-11-30

**Authors:** Roland Grad, Pierre Pluye, Janique Johnson-Lafleur, Vera Granikov, Michael Shulha, Gillian Bartlett, Bernard Marlow

**Affiliations:** ^1^Information Technology Primary Care Research GroupDepartment of Family MedicineMcGill UniversityMontreal, QCCanada; ^2^College of Family Physicians of CanadaMississauga, ONCanada

**Keywords:** Electronic mail, clinical email channels, information retrieval, physicians, family

## Abstract

**Background:**

A synopsis of new clinical research highlights important aspects of one study in a brief structured format. When delivered as email alerts, synopses enable clinicians to become aware of new developments relevant for practice. Once read, a synopsis can become a *known item* of clinical information. In time-pressured situations, remembering a known item may facilitate information retrieval by the clinician. However, exactly how synopses first delivered as email alerts influence retrieval at some later time is not known.

**Objectives:**

We examined searches for clinical information in which a synopsis previously read as an email alert was retrieved (defined as a *dyad*). Our study objectives were to (1) examine whether family physicians retrieved synopses they previously read as email alerts and then to (2) explore whether family physicians purposefully retrieved these synopses.

**Methods:**

We conducted a mixed-methods study in which a qualitative multiple case study explored the retrieval of email alerts within a prospective longitudinal cohort of practicing family physicians. Reading of research-based synopses was tracked in two contexts: (1) push, meaning to read on email and (2) pull, meaning to read after retrieval from one electronic knowledge resource. Dyads, defined as synopses first read as email alerts and subsequently retrieved in a search of a knowledge resource, were prospectively identified. Participants were interviewed about all of their dyads. Outcomes were the total number of dyads and their type.

**Results:**

Over a period of 341 days, 194 unique synopses delivered to 41 participants resulted in 4937 synopsis readings. In all, 1205 synopses were retrieved over an average of 320 days. Of the 1205 retrieved synopses, 21 (1.7%) were dyads made by 17 family physicians. Of the 1205 retrieved synopses, 6 (0.5%) were known item type dyads. However, dyads also occurred serendipitously.

**Conclusion:**

In the single knowledge resource we studied, email alerts containing research-based synopses were rarely retrieved. Our findings help us to better understand the effect of push on pull and to improve the integration of research-based information within electronic resources for clinicians.

## Introduction

The environment of primary care medicine severely limits time for searches of clinical information. At the point of care, and given the time required for searches, using electronic knowledge resources during the consultation is perceived to be a complex task [1]. Away from the point of care, keeping up with the literature involves selecting and interpreting relevant clinical research, which is far from trivial.

**Figure 1:**
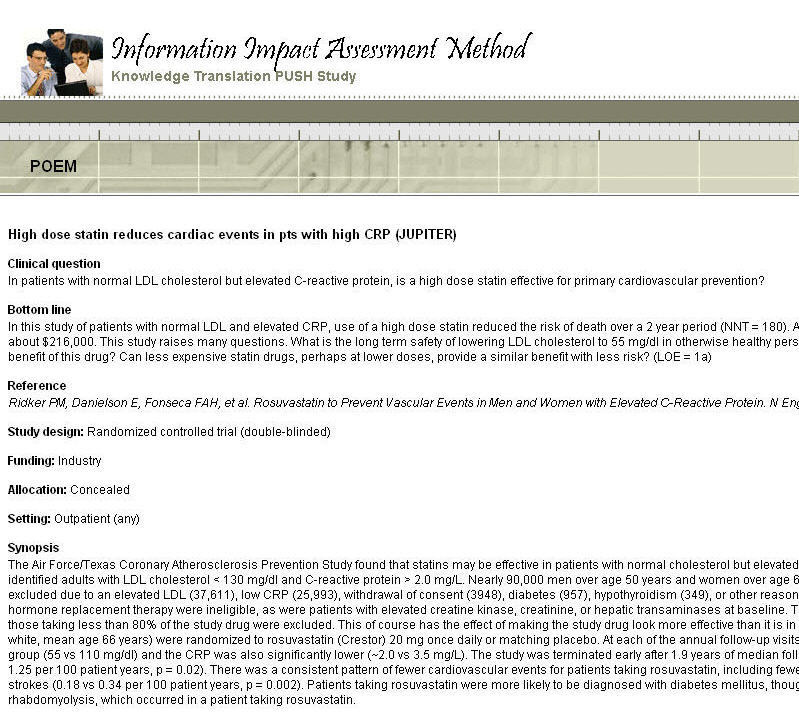
Example of a synopsis.

Reading synopses of new clinical research delivered as email alerts allows clinicians to become aware of new developments relevant for practice [2-4]. A synopsis consists of important aspects of a research study presented in a brief structured format that allows for quick reading (see Figure 1). These synopses are often emailed on a daily or weekly basis [5]. To facilitate retrieval of synopses first delivered as email alerts, some electronic knowledge resources make these synopses available within searchable databases [6]. One example of such integration is Essential Evidence Plus featuring POEMs (patient-oriented evidence that matters) [7].

Our literature review of email alerts in clinical practice found only five evaluation studies in the health sciences [8-12]. Citation tracking of these papers and a subsequent literature search resulted in one study. This study demonstrated that email to adults from rural counties containing short updates of new content on a nutrition website increased usage of that website [13]. Outside of medicine, marketing research and business literature have long noted the ability of targeted and personalized email to increase traffic to websites, increase sales and revenue, and create an interactive relationship with the recipient [14-16]. In information science, the concept of known items and known-item searching has been explored since the early 1980s. It has been demonstrated that users of online library catalogs are more likely to be successful when searching for a known item as opposed to a more general subject search [17]. 

Previously, we have proposed a “push-pull” conceptual framework [18]. In this framework, it is assumed the *push* of clinical information will stimulate *pull* through the retrieval of objects of pushed information. In medicine, one study has examined the effect of push on pull [10]. In a cluster randomized trial of McMaster PLUS software, 203 physicians used either a full-serve version (that included email alerts to new articles and a cumulative database of email alerts) or a self-serve version that included the database and a passive guide to evidence-based literature. On average, physicians receiving the full-serve version made 0.77 more log-ins per month. How email alerts modestly increased log-ins to McMaster PLUS software was not reported. Thus, we do not know how push may influence pull in terms of retrieval of objects of pushed information.

Given the demands of practice and the limits of human memory, we assumed clinicians would occasionally need to retrieve information they had previously read as an email alert. Once read, email alerts can become known items of information. A search for a known item may include the author, the title, the subject, or a combination of these and other information [19]. If the push of synopses led to the creation of known items, retrieval of this information would be facilitated, helping to meet the demands of clinical practice in time-pressured situations. In addition, knowing about a synopsis might overcome one of the most common reasons given by physicians for not pursuing a clinical question—doubt about the existence of relevant information [20]. Therefore, we conducted a study of how the push of synopses of clinical research can lead to their subsequent retrieval by family physicians. We did this by prospectively identifying push-pull events operationalized as dyads. A dyad was defined as an occurrence of a family physician retrieving a synopsis from a knowledge resource when that synopsis had been read previously as an email alert.

Our study objectives were to (1) examine the retrieval of synopses from a knowledge resource among family physicians reading synopses as email alerts and then (2) using brief interviews, explore whether family physicians purposefully retrieved synopses that had been previously read as email alerts. 

## Methods

### Study Design and Participants

A mixed-methods study was conducted using a *validation* design [21]. A qualitative multiple case study explored results from a prospective longitudinal cohort. We chose an exploratory naturalistic approach given that we did not know either the frequency or the variety of reasons why physicians retrieve synopses they previously read as email alerts. From 9 of 10 provinces, 41 family physicians consented to participate. Of these, 36 were certificants of the College of Family Physicians of Canada (CFPC). There were 24 men and 17 women ranging in age from 28 to 70 years (median 44 years). In addition, of these 41 family physicians, 28 (68%) had a faculty appointment, and all were in active practice. With respect to their main patient setting, 1 family physician had no Internet access, 37 (90%) reported having high-speed access, and 3 did not know what type of connection they had. In terms of computer self-efficacy, 8 (20%) rated their level of skill as advanced, 32 (78%) as intermediate, and 1 as beginner. Early on, 1 participant dropped out of the study before retrieving any synopses. The study protocol was approved by the McGill University Faculty of Medicine Institutional Review Board.

### Quantitative Methods With Respect to Objective 1: Do Family Physicians Retrieve Synopses They Previously Read as Email Alerts?

#### Data Collection

We maintained two separate websites (push and pull). Using a method described elsewhere, we pushed titles of newly released POEMs, hereafter referred to as synopses, to participants by email on weekdays beginning January 7, 2008 [12]. Participants only read synopses they wished to read after clicking on a link in the email message. Ratings of these emailed synopses were also collected at our push website. Ratings were made using the Information Assessment Method (IAM) (described below), and participants earned continuing education credits for this activity, which has been accredited in Canada since 2006. This method, IAM, is a product of our funded research program [22].

To enable and track retrieval of these synopses, each participant received a handheld computer, that is, a personal digital assistant (PDA) or Smartphone containing Essential Evidence Plus. We performed the initial software installation so the device was ready to go on delivery. We specifically chose the PDA for several reasons. First, as a single-user device, a PDA facilitates data collection by attributing information hits to one user. Second, many family physicians are willing to use PDA software for addressing questions arising in their practice. While all participants were offered the HTC Touch Smartphone, 17 chose a PDA with no phone, the hp iPAQ 110. All devices ran the Windows Mobile 6 operating system and were Wi-Fi enabled. However, no data plan was provided and PDA software was used offline. 

**Figure 2:**
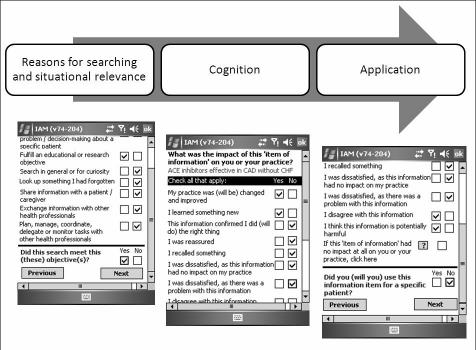
Questionnaire from Information Asessement Method (IAM) linked to one search in Essential Evidence Plus.

On each PDA, IAM integrated with Essential Evidence Plus to track all opened information hits as well as the date and time of each search. Using a checklist of seven reasons, IAM prompted each participant to report the reason for their search [23]. IAM then asked the participant to rate the retrieved information in relation to three constructs: (1) situational relevance, (2) cognitive impact, and (3) use of the retrieved information for a specific patient. Figure 2 below shows screen shots from the IAM questionnaire and their corresponding constructs. Participants were trained to use Essential Evidence Plus, and their IAM ratings were transferred to our pull website when their PDA synced with their personal computer (PC). Participants entered the study from November 2007 through May 2008. Each participant had a unique start date defined by the date of their first rated search. Data collection ended in March 2009.

#### Data Analysis

With respect to our first objective, the reading of synopses was tracked in push and pull. Each read synopsis was date and time stamped and attributed to a specific participant. All retrieved synopses previously read as an email alert were classified as dyads, regardless of whether that synopsis was rated. We calculated the number of dyads in total and by participant as well as the time to their occurrence based on the date the synopsis was first read on email. 

### Qualitative Methods With Respect to Objective 2: Do Family Physicians Purposefully Retrieve Synopses Previously Read on Email

#### Data Collection

With respect to our second objective, each dyad was a case. On a weekly basis, push and pull databases were merged to identify dyad occurrences. When a dyad was identified, an interview was scheduled and conducted by author JJL. Interviews were recorded on audiotape and transcribed verbatim. Brief semistructured telephone interviews (lasting 16 minutes on average) were conducted from March 2008 through February 2009. The average time from dyad occurrence to interview was 31 days (range 4-110 days). A longer time to interview was explained by a number of factors, such as delays in synchronizing the handheld computer for data transfer.

Even though we had the dyad concept in mind, interviews were exploratory and began with an open-ended screening question, “Do you remember why you retrieved this POEM?” The purpose of these questions was to identify dyad-related searches that were clearly remembered. This exploratory approach also allowed us to uncover other reasons why a dyad occurred. If the physician’s memory of the reason for searching was unclear, the interview ended.

Guided by their personal portfolio of synopsis ratings (quantitative data), which served to remind the participant of the context around the retrieval of specific synopses, interviewees recounted their story around the search. The interview focused on (1) why the search was done, (2) the cognitive impact of information they retrieved, and (3) any application of that information for a specific patient. They were also questioned about perceived patient outcomes. (Our interview guide is available on request.)

#### Data Analysis

We defined the concept of known items in line with Allen’s description in which a user is trying to find an item previously read [24]. Qualitative data consisted of synopses that were read, documents (interviewees’ portfolios including ratings and free-text comments on synopses), field notes, and interview transcripts. A thematic analysis was conducted [25]. Text files of transcribed interviews were imported into specialized software (NVivo7, QSR International, Victoria, Australia). Extracts of interviews were assigned by two of the authors (PP and JJL) to emerging themes as suggested by the data. Based on these themes, initially there were three types of dyad: (1) known item, (2) serendipitous, and (3) critical thinking. After group discussion, initial dyad types were refined and organized into two categories (purposeful and serendipitous), each with two subcategories (1a) purposeful, known item, (1b) purposeful, critical thinking, (2a) serendipitous, recognized when reread, and (2b) serendipitous, not recognized when reread. The difference between purposeful and serendipitous information retrieval can be described as follows. In contrast to purposeful retrieval, in a serendipitous encounter the user finds information *not by intention* and the existence or location of information is unexpected [26,27].

For each dyad, five researchers independently assigned interview extracts to dyad types as suggested by the data. These assignments consisted of an iterative process until consensual understanding was achieved. Disagreements were resolved by discussion during consensus meetings.

## Results

### Quantitative Results With Respect to Objective 1

In the push component, participants had the opportunity to read and rate 194 synopses delivered from January 7, 2008, through December 12, 2008 (or 7814 total opportunities). In this 341-day time window, we documented 4937/7814 (63%) synopsis readings and 4548/7814 (58%) synopsis ratings. On average, 111 synopses were rated per participant (range 11 to 189 ratings). No cognitive impact was reported in 1018 synopsis ratings, while 3530 synopsis ratings contained one or more types of cognitive impact. These ratings are summarized in Table 1.

**Table 1:** Push: Reports of cognitive impact by type

Type of cognitive impact^a^	n
I learned something new.	2543
I was reassured.	1637
I am motivated to learn more.	1570
This information confirmed I did (will do) the right thing.	1419
This information had no impact at all on me or my practice.	1018
I am reminded of something I already knew.	942
My practice was (will be) changed and improved.	922
I am dissatisfied, as there is a problem with this information.	258
I am dissatisfied, as this information has no impact on my practice.	126
I think this information is potentially harmful.	65
I disagree with this information.	37

^a^More than one type of cognitive impact could be reported for each synopsis.

In the pull component, searches were tracked over a mean of 320 days of follow-up (range 43 to 428 days). We documented 2170 searches in Essential Evidence Plus, and in these searches, 1205 synopses were retrieved. Participants’ reasons for searching are reported in Table 2.

**Table 2:** Participants' reasons for searches for clinical information

Reason for searching^a^	n
Address a clinical question, problem, or decision about a specific patient	1310
Look up something I had forgotten	672
Share information with a patient/caregiver	624
Exchange information with other health professionals	520
Search in general or for curiosity	496
Fulfill an educational or research objective	434
Plan, manage, coordinate, delegate, or monitor tasks with other health professionals	197

^a^More than one reason could be reported per search.

**Figure 3:**
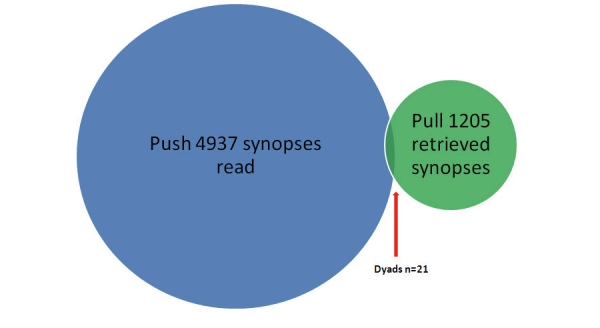
Number of dyads in the context of synopses read in push and subsequently retrieved. Where dyad signifies one participant retrieving one synopsis he or she previously read on email.

Of the 1205, 21 (1.7%) retrieved synopses were dyads made by 17 participants. Of these, 13 participants made 1 dyad, 3 participants were responsible for 2 dyads each, and 1 participant made 3.

### Qualitative Findings With Respect to Objective 2

All 17 participants were interviewed about their dyads, a detailed example of which is presented as Multimedia Appendix 1. Of the 17 participants, one did not clearly remember the dyad.

#### Purposeful, Known Item Dyads

Of 21 dyads, 6 (28*%)* were concordant with the known item type of dyad, defined as a search for one synopsis previously read on email. By way of illustration, a participant read and rated a synopsis entitled “Single dose of honey effective for cough in kids” and stated:

I wanted to have a copy…for teaching purposes….Well I knew it existed [the synopsis]. When I first read it, I did not write where the article was from to be able to retrieve it. So I had to retrieve it to find which journal it was in

This extract was interpreted as a known item since the synopsis was retrieved by the participant on purpose because they knew it existed.

#### Purposeful, Critical Thinking Dyads

Of 21 dyads, 4 (19%) were concordant with the critical thinking type of dyad, defined as a subject search triggered by the content of one pushed synopsis leading to retrieval of other information including that synopsis. In line with Mitchell et al, critical thinking refers to questioning the credibility of clinical information—that is, the accuracy or trustworthiness of clinical information [28]. For example, a participant read one synopsis entitled “Liquid-based equals conventional cervical cytology” and stated:

I read that synopsis, and I was very surprised. So I went looking for more information on Pap smears, how accurate they were and more evidence-based material. I did that through Essential Evidence Plus and through Google. And then I went back to review that synopsis, to make sure I understood what I had read

This extract suggests the participant was surprised by the content of the pushed synopsis, and this surprise prompted a search. During the search, the participant then retrieved the same synopsis on PDA.

#### Serendipitous Dyads, Recognized When Reread

Of 21 dyads, 3 (14%) were concordant with our proposed subcategory, serendipitous dyad, recognized when reread. This subcategory is defined as a synopsis retrieved during a subject search on a related topic and clearly recognized when reread. For example, a participant read a synopsis entitled “OCs not associated with overall cancer risks” and stated:

I did a search on the oral contraceptive pill….It was an educational sort of thing I wanted to do for myself....A case came up in the office about birth control....At the time I had forgotten that I had read it [the synopsis]....Then, when I actually read it, I recognized it was something I had read previously

#### Serendipitous Dyads, Not Recognized at All

Of 21 dyads, 7 (33%) were concordant with our proposed serendipitous dyad, not recognized at all, defined as a synopsis retrieved in a subject search on a related topic but not clearly recognized when reread or not recognized at all. By way of illustration, a participant read a synopsis entitled “Breastfeeding does not decrease asthma/allergy” and stated:

I was actually looking for some information because I did have a patient who asked me about breastfeeding and allergy…. No, I don’t remember [having previously read this synopsis on email].

### Findings From Mixing Quantitative and Qualitative Data

Critical thinking dyads are unique in so far as they occurred on the same day that the emailed synopsis was read. No pattern is apparent with respect to the timing of the other dyad types, as shown in Table 3.

**Table 3:** Number of dyads by type and time of occurrence

Type of Dyad	Time Interval Between Reading in Push and Pull (Days)
Purposeful dyads, known item (n = 6)	0 to 323
Purposeful dyads, critical thinking (n = 3)	Same day
Serendipitous dyads, recognized when reread (n = 3)	22 to 87
Serendipitous dyads, not recognized (n = 5)	19 to 317
Excluded dyad, forgotten (n = 1)	106

## Discussion

**Figure 4:**
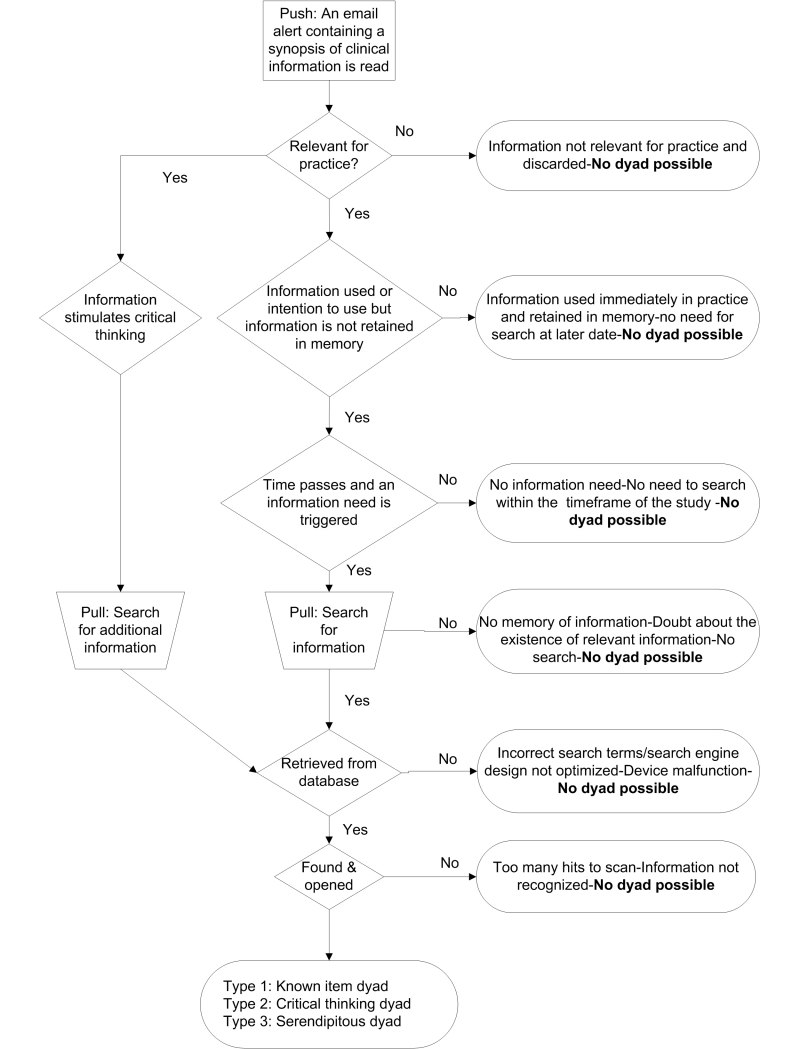
Influence of push on pull: Why dyads are so rare.

In clinical medicine, how the push of synopses of clinical research leads to their retrieval is examined in this study. In two situations (known item and critical thinking), family physicians purposefully retrieved a synopsis they had previously read as email. Although the combination of quantitative results and qualitative findings suggests dyads are rare events representing a very small proportion of retrieved information, their occurrence supports our push-pull framework. The rarity of dyads arises from a range of contributing factors outlined in the flow diagram shown in Figure 4.

The value of linking the push with the pull of research-based information for practice has been proposed [29-31]. However, our literature review and findings from this study reveal that push and pull are largely treated as separate but important processes. This separation of push and pull can paradoxically complicate the use of clinical information in practice. For example, within a typical primary care patient visit, a known-item search for a synopsis about the dose of metformin for prevention of type 2 diabetes yields such a large set of results that the clinician cannot locate the “needle in the haystack.” Our findings suggest a need for a simple method to permit physicians to label a synopsis as a favorite. This would facilitate the creation of user-specific subsets of favorite synopses. Searches for known items within these subsets would be less time consuming and more successful than searching an entire database. In at least one clinical resource, users can presently save synopses delivered as email alerts in a favorites list [32]. 

Built over years of training and experience, physician memory of clinical information is a critical aspect of any search for known items. In addition to brief reading of clinical information, interactions with colleagues, local opinion leaders, and pharmaceutical representatives are experiences shared by many physicians. In theory, a time-pressured physician needs an efficient search strategy such as known-item searching. However, the capacity to conduct known-item searching is dependent on long-term memory of a specific object of information. Long-term memory can be roughly divided into episodic, semantic, and procedural memory [33]. Semantic memory is the memory of our general knowledge about the world and includes remembering specific information such as facts derived from reading text. When looking for information, semantic memory is called upon for known items of information such as synopses read on email. However, a single exposure to one synopsis on email is a small stimulus to memory, especially as each new day brings the delivery of one new synopsis. Thus, over time, factual knowledge derived from brief reading of email alerts of synopses may be simply forgotten. In related work on cognitive processing and memory, we found the ability of family physicians to remember synopses they previously read declined over time [34]. Future research should strive to help us better understand if the low level of dyads we observed in this study is related to memory, search skills, limitations of push technology, search engine design [35], or simply low demand for clinical information about problems rarely encountered in primary care practice.

### Limitations

Our work faced sociotechnical limitations. For example, we could not track failed known-item searches or events where participants retrieved a known synopsis from a database other than Essential Evidence Plus on their PDA. In other work, we interviewed physicians who rated synopses they received on email, similar to the push component of this study. In our other work, we found that of 46 physicians, 8 (17%) said they retrieved synopses as archived email, 3 (7%) said they used a database other than Essential Evidence Plus for retrieving synopses, while 1 (2%) printed synopses for rereading [36]. In the current study, some participants reported technical problems with their PDA, making it likely that searches for synopses were occasionally done at a PC workstation rather than on their PDA. Searches done at a PC workstation could not be tracked [37]. All of these factors reduced our ability to document the occurrence of dyads.

On the other hand, two-thirds of our study cohort were family physicians involved in teaching students or residents. Thus, unlike other studies that excluded academic physicians [38], our data were obtained from a select group who were motivated to read and retrieve synopses for teaching or rhetorical purposes. The motivation to read synopses on email and to search for synopses in one handheld knowledge resource in the context of a research study likely increased the frequency of occurrence of dyads. For some participants, rating a POEM may have enhanced memory of that POEM, and semantic memory is a prerequisite for a known item dyad. For other participants, receiving a PDA may have contributed to a Hawthorne effect that influenced the frequency of their searching.

A strength of our mixed-methods study resides in the integration of qualitative findings and quantitative data to examine the push and pull of research-based synopses. First, the quantitative component allowed us to identify rare dyads in the midst of a large number of information delivery and retrieval events. Prospective identification of dyads through careful tracking allowed us to conduct interviews guided by participants’ rating of synopses. Finally, the qualitative component provided some understanding of how participants experienced these events. 

### Conclusion

In conclusion, email alerts of research-based synopses were rarely retrieved. Our findings help us to better understand the effect of push on pull and to improve the integration of research-based information within electronic resources that are increasingly used by clinicians.

## Multimedia Appendix 1

Example of a purposeful known item dyad.

## Abbreviations

CFPC: College of Family Physicians of Canada
IAM: Information Assessment Method
PC: personal computer
PDA: personal digital assistant
POEMS: patient-oriented evidence that matters
