# Supplementary material for: Do Family Physicians Retrieve Synopses of Clinical Research Previously Read as Email Alerts?
Source: J Med Internet Res. 2011 Nov 30;13(4):e101. doi: 10.2196/jmir.1683 (PMC3278087; doi:10.2196/jmir.1683)
Supplement: Supplementary file 1 [file jmir_v13i4e101_app1.pdf]

On July 28 2008, Dr Smith read and rated an InfoPOEM on email entitled "*ABCD score predicts seven day stroke risk in patients with TIA*". This InfoPOEM had the following types of cognitive impact: *learning*, *confirmation*, *reassurance* and *motivation to learn more*.

Then on December 11 2008, Dr Smith was working in the emergency department. In the presence of a trainee, and during a patient encounter, he searched for information on transient ischemic attack. The same InfoPOEM "*ABCD score predicts seven day stroke risk in patients with TIA*" was retrieved to address a clinical question, to look up something he forgot, and to share information with a patient and with other health professionals. *"I was [looking for that particular InfoPOEM] actually […] I retrieved it while I was working in the emergency department […] I was seeing a patient with TIA symptoms […] I remember discussing it [the InfoPOEM] with a resident or a student[…]The reason for the search was to have good data to discuss the level of urgency with which the patient should be seen by a neurologist […] It's sometimes difficult to get them [neurologists] to see the patient "sur place", immediately […] So it was just to be well informed. It would give me some additional ammunition to have the neurologist come in and see the patient."*

When evaluated in the search context, the InfoPOEM had the following types of cognitive impact: *recall*, *confirmation*, and *reassurance*. "*The information confirmed what I think… there is consensus in emergency practice literature that this is the right thing to do […] [And] I had read the POEM before so I remembered it."*

Dr Smith used this information to maintain and justify the management of the patient, a woman in her seventies. "*The neurologist saw the patient. The patient had a CT scan. The patient was put on medication and discharged for outpatient follow-up for other studies. But fairly expeditiously, so…investigations were done on short-order."*

Finally, no patient health outcomes were associated with the application of this information. "*Had I not retrieved this POEM, things would have proceeded exactly as they had […] [But] in another context, there might be a patient that was more borderline and maybe a neurologist who was less compliant where it might have made a difference, it might have [helped] to persuade the neurologist to see the patient.*"
